# The Metric System

**Published:** 1880-08

**Authors:** T. W. O’Kelly

**Affiliations:** Conyers, Ga.


Medical and Surgical Journal.

VOL. XVIII.] AUGUST—1880. [No. 5.

### Original Communications.

### THE METRIC SYSTEM.

BY T. W. O'KELLY, M.D., CONYERS, GA.

"As you have always been on the alert for the advancement of medical science, and have always favored advancement in other branches, according to your motto, "*Pax et Scientia sed Veritas sine Timore*," I would ask space in the JOURNAL for a few reasons in answer to "The Objections to the Metric System," found in the June number.

The first trouble comes from the employment of "Latin and Greek terms." It does seem that after one has learned the names of all the bones, muscles and nerves of a human body, he would not stagger at learning three French, three Greek and three Latin terms to denote the whole Metric System, including weight, length and capacity measure; French denoting the kind of measure, Greek the multiple and Latin the decimals ot the common unit. It is as simple as it can be and be scientific. Nor do we have to learn these foreign languages before we can comprehend the meaning of the terms used, any more than we do to comprehend the meaning of grain, scruple, drachm, ounce, pound and others, coming as they do, from one of those languages. So we see no disadvantage in this.

We claim more than one advantage for the decimal Metric System. In the first place, it is at once more comprehensive and more accurate than the old system ; for there is no necessary limit either in going up or down from what is usually called the unit. As a proof of its accuracy, it is used by all good chemists, a class of scientists necessarily much more accurate than physicians are, even in the dispensation of poison.

Secondly, the carrying unit being always ten, quantities are more easily reduced from one denomination to another, by simply moving the decimal point to the right or to the left. It is true, that there would be some trouble in reducing "fractional cubic feet," to their places in the table; but this is no argument against the system itself, for the same trouble would be experienced in reducing from the new system to the old.

Thirdly, quantities may be expressed with fewer figures and in a form more easily comprehended by the new than by the old. Take for example :

| New System. | | Old System. |
|---|---|---|
| 1 cgm. | = | 1-6 gr. |
| 2.37 gms. | = | 1℈ 17 5-6 grs. |
| 10 mgm. | = | 1-600 gr. |

There is no need of expressing everything in the denomination of gramme — take a smaller. Are those above too small ? Then there should be no complaint about inaccuracy. Nor are a great many figures required to express any quantity, except a large amount very accurately. One could have such a weight as 168.3674. Those who have curiosity enough may see what this would be in drachms, etc. It would be no better.

Now let us see how it would do in posology. Still taking from the article in June number, we have :

| Remedies. | Old System. | | New System. |
|---|---|---|---|
| Gum. Opii............ | gr. ½ to ii. | = | 3 to 6 cgms. |
| Aconitia........ | gr. 1-200 to 1-50. | = | 1-5 to 1 mgms. |
| Ferri chor. tinct... | gtts x to xxx. | = | 0.6 to 1.8 c.c. |

I appeal to the unbiased judgment of any one equally acquainted with both to say which is the most easily comprehended. If the figures are lengthened more than necessary in a dose book, the fault is in the author, not in the system, just as in reduction.

One of the great advantages as well as beauties of the new, is that any denomination can be taken for the unit without the least trouble. If I knew more of medicine perhaps I could see why men of medicine should not use it, and as it is, certainly I can not.

But while we see no objections to the system itself, we must admit that some might, with reason, be urged against the change. Were the change sudden, those who have scales or balances should be compelled to lay by the old and buy new ones; the new tables would be to learn, and considerable labor and trouble would be required to reduce the many old records to the their new equivalents.

Still in spite of all these difficulties, the Metric is the only system we can hope to become universal—that is, belonging to all classes. Would this not be better? Why should the merchant have one kind of weights, the jeweler another, the apothecary another, and the chemist still another? No good reason, but so it is; and no one can understand the weights of the others without special study.

So long as there are young men under my instruction they shall be taught, as far as practicable, the advantages of the Decimal Metric System. I hope that men of science, medical included, will push forward this movement to improve the weights and measures of this country. They are the only ones who can do it. The change must come, but gradually.

